# Sexual selection reinforces a higher flight endurance in urban damselflies

**DOI:** 10.1111/eva.12485

**Published:** 2017-05-11

**Authors:** Nedim Tüzün, Lin Op de Beeck, Robby Stoks

**Affiliations:** ^1^ Laboratory of Aquatic Ecology, Evolution and Conservation University of Leuven Leuven Belgium

**Keywords:** eco‐evolutionary dynamics, generalized multilevel path analysis, geometric morphometrics, habitat fragmentation, rapid evolution, scrambling competition

## Abstract

Urbanization is among the most important and globally rapidly increasing anthropogenic processes and is known to drive rapid evolution. Habitats in urbanized areas typically consist of small, fragmented and isolated patches, which are expected to select for a better locomotor performance, along with its underlying morphological traits. This, in turn, is expected to cause differentiation in selection regimes, as populations with different frequency distributions for a given trait will span different parts of the species’ fitness function. Yet, very few studies considered differentiation in phenotypic traits associated with patterns in habitat fragmentation and isolation along urbanization gradients, and none considered differentiation in sexual selection regimes. We investigated differentiation in flight performance and flight‐related traits and sexual selection on these traits across replicated urban and rural populations of the scrambling damselfly *Coenagrion puella*. To disentangle direct and indirect paths going from phenotypic traits over performance to mating success, we applied a path analysis approach. We report for the first time direct evidence for the expected better locomotor performance in urban compared to rural populations. This matches a scenario of spatial sorting, whereby only the individuals with the best locomotor abilities colonize the isolated urban populations. The covariation patterns and causal relationships among the phenotypic traits, performance and mating success strongly depended on the urbanization level. Notably, we detected sexual selection for a higher flight endurance only in urban populations, indicating that the higher flight performance of urban males was reinforced by sexual selection. Taken together, our results provide a unique proof of the interplay between sexual selection and adaptation to human‐altered environments.

## 1 INTRODUCTION

Urbanization is a major human‐induced selective force driving rapid phenotypic change and evolution (Alberti, 2015; Alberti et al., 2017; Sullivan, Bird, & Perry, 2017). Urbanized areas typically consist of small, fragmented and isolated patches (Parris, 2016). In addition, urban roads with heavy traffic can act as strong barriers to the movement of many organisms, including flying insects (Muñoz, Torres, & Megías, 2015). As a result, urban habitats will likely not be colonized by a random set of immigrants from rural populations (e.g., Evans et al., 2009). Surprisingly few studies, however, considered differentiation in phenotypic traits associated with patterns in habitat fragmentation and isolation along urbanization gradients (but see Cheptou, Carrue, Rouifed, & Cantarel, 2008; San Martin y Gomez & Van Dyck, 2012; Schoville, Widmer, Deschamps‐Cottin, & Manel, 2013; Dubois & Cheptou, 2016).

One trait frequently studied in relation to habitat fragmentation and isolation in general is dispersal ability (Baguette, Legrand, Fréville, Van Dyck, & Ducatez, 2012; Cote et al., 2017). Traits related to dispersal ability such as locomotor performance (e.g., Phillips, Brown, Webb, & Shine, 2006) and its underlying morphological traits are usually increased in fragmented and isolated populations (Baguette et al., 2012; Van Dyck & Matthysen, 1999). However, depending on the spatial resource distribution, mobility can also be reduced in fragmented landscapes (Bergerot, Merckx, Van Dyck, & Baguette, 2012). Colonization of empty patches is a process tightly associated with dispersal ability. Consequently, similar findings have been reported in studies comparing edge and core populations of range expanding species: increased dispersal ability is common in the more recently colonized edge populations (Hill, Griffiths, & Thomas, 2011). Both spatial sorting (Shine, Brown, & Phillips, 2011), the process where only the organisms with the best locomotor abilities end up at the range front, and local adaptation (Travis & Dytham, 2002) may contribute to dispersal‐enhancing phenotypes at invasion fronts, although spatial sorting has been suggested as the main driver (Van Petegem, Boeye, Stoks, & Bonte, 2016). Considering that urban habitats are colonized by rural source populations (e.g., Evans et al., 2009), and given the typically fragmented and isolated urban habitats (e.g., Cane, Minckley, Kervin, Roulston, & Neal, 2006; Luck & Wu, 2002), only the best dispersers from the rural populations are likely to enter urban habitats. Therefore, we could expect an increased locomotor performance and associated phenotypic traits in urban populations.

Changes in phenotypes caused by evolution may generate feedback loops from evolution to ecology (Hendry, 2016). One such important but relatively understudied by‐product of differences in mean phenotypic traits between populations is its effect on the direction and strength of selection. Populations with different frequency distributions for a given trait will span different parts of the species’ fitness function. When the fitness function of the species is nonlinear, this will result in different selection patterns in the different populations (Endler, 1986; Figure 6.5 in Conner & Hartl, 2004). Therefore, the human‐induced changes in mean phenotypic traits in urban habitats (Alberti et al., 2017; Sullivan et al., 2017) can be expected to result in different selection regimes between urban and rural populations. This may be especially true for sexual selection in mating systems such as scrambling competition where increased locomotor performance is selected for (Husak & Fox, 2008). We therefore hypothesize the higher mean values of locomotor traits that are to be expected in urban populations to generate different sexual selection patterns on these traits.

We investigated differentiation in flight performance and flight‐related traits and sexual selection on these traits between urban and rural populations of the scrambling damselfly *Coenagrion puella*. Due to their conspicuous mating behaviour localized at pond margins, damselflies are ideal study organisms to study sexual selection in natural populations (Córdoba‐Aguilar, 2008). We quantified flight performance (flight endurance and flight speed) and measured a set of flight‐related phenotypic traits including wing morphology and physiology (relative fat content and flight muscle ratio) of mated and unmated males. To reveal the covariation patterns between the flight‐related traits, flight performance and mating success and whether these differed between urban and rural populations, we used a path analysis approach, a powerful tool to investigate sexual selection (Kingsolver & Schemske, 1991). We expected sexual selection for a higher flight endurance, as this increases the chances of encountering mates in scrambling species (Husak & Fox, 2008). In line with this, we expected increased investment in morphological and physiological traits enhancing flight performance in mated males, although costs associated with mating may obscure patterns of sexual selection on these traits (e.g., energy reserves: Blanckenhorn, Kraushaar, & Reim, 2003; Blanckenhorn, Kraushaar, Teuschl, & Reim, 2004). Based on the characteristics of urban habitats discussed above (Parris, 2016), we predicted urban males to show a higher flight performance and associated phenotypic traits compared to rural males. Given that sexual selection patterns can be shaped by mean phenotypic trait values (Endler, 1986), we predict differentiation in sexual selection on flight performance between urban and rural populations. Given the expected higher flight endurance in urban populations, and the finding that flight endurance only positively influences mating success above a threshold value in the study species (Gyulavári, Therry, Dévai, & Stoks, 2014), we predicted stronger sexual selection on flight endurance in urban populations.

## 2 MATERIALS AND METHODS

### 2.1 Study populations and sampling

*Coenagrion puella* is one of the most common damselflies in Europe (Dijkstra & Lewington, 2006), occupying ponds in both rural and urban areas (Goertzen & Suhling, 2013). We studied three rural populations (Bierbeek, Bornem and Houwaart) and three urban populations (Leuven, Mechelen and Oudenaarde). All six populations were situated within a 45‐km radius in Flanders, Belgium (Table S1, Fig. S1). We used a two‐step procedure using geographic information system (GIS) for the selection of urban and rural ponds. First, we selected three urban plots with >15% built‐up area, and three rural plots with <3% built‐up area, all 3 × 3 km. Next, we selected a subplot of 200 × 200 m in each plot, with the same urbanization level following the same criteria of percentage built‐up area. This way we made sure that both the direct environment, represented by subplots, and the broader surroundings, represented by plots, reflected the same urbanization level. This sampling design was also applied in a recent study by Piano et al. (2017).

Males of *C. puella* search for females by patrolling low at the breeding pond and display scramble competition for mates. Female choice behaviour is thought to be not important in this species (Banks & Thompson, 1985). To assess sexual selection on traits, we sampled mated and unmated males in all six populations during July 2013. Comparing trait values between sets of mated and unmated males is an often used method to detect sexual selection (e.g., Blanckenhorn, Morf, Mühlhäuser, & Reusch, 1999; Gosden & Svensson, 2008). We categorized males as mated when they were caught in tandem position or copulation. Unmated males were those not associated with a female but that were active at the reproduction site. As the average duration of association between a couple is ca. 2 hr (Banks & Thompson, 1985), and we collected all individuals between 11:00 and 15:00 hr when sexual activity peaks, we lowered the probability of wrongly categorizing a male as unmated that would have mated that day. Yet, we cannot fully exclude that we might have missed matings on a given day and that our sample of “unmated” males contained males that would have mated that day. Nevertheless, this would make our results of sexual selection conservative as it would introduce noise in the data set and make it harder to detect phenotypic differences between our samples of mated and unmated males. Despite this limitation, this technique has been successfully applied to detect signals of sexual selection in damselflies (e.g., Gosden & Svensson, 2008), including the study species (Gyulavári et al., 2014). In total, 576 males (292 mated, 284 unmated) were collected on sunny days near the border of their breeding pond (Table S1). All males were transferred individually to the laboratory in 50‐ml plastic cups (diameter: 3.5 cm; height: 7 cm) and were maintained in an incubator at 14°C until the start of the flight test. To control for potential effects of the time span between capture and the flight test, we recorded the time of capture of each individual.

### 2.2 Flight test

We tested the flight performance (flight speed and flight endurance) of individuals following the methodology used before in damselflies (Gyulavári et al., 2014, 2017; Therry, Gyulavári, Schillewaert, Bonte, & Stoks, 2014). Flight performance traits were quantified when animals were flying in a plexiglass flight tube (diameter: 50 cm; height: 200 cm) in a temperature‐controlled room (21.5 ± 0.5°C). The individual to be tested was placed in its cup at the bottom of the flight tube and was allowed to acclimatize for 3 min. To initiate the flight test, the cup was gently rotated horizontally until the individual took off. All flight tests were conducted on the day of capture.

A typical flight bout consisted of an initial, fast and linear upward trajectory, followed by a slower trajectory where the individual stepwise ascended and eventually reached the highest vertical distance of its flight trajectory. The frictionless surface of the tube prevented individuals from resting on the tube walls. Hence, a flight bout ended when the individual landed on the bottom of the flight tube. We recorded the following parameters during each flight bout: (i) the height reached during the initial trajectory (“initial height”), (ii) the duration of this initial trajectory (“initial duration”) and (iii) the total duration of the flight bout (“total duration”). The flight tube was graded every 10 cm to allow estimating the height parameters. The flight durations were measured using a chronometer (accuracy 0.01 s). We estimated flight speed as “initial height”/”initial duration”, whereas “total duration” was interpreted as flight endurance. Each individual was tested only once. Flight performance tested this way has been shown to be repeatable in another damselfly (Gyulavári et al., 2017) and to be related to mating status in the field (Gyulavári et al., 2014; Therry et al., 2014).

To correct for potential influences of body temperature on flight performance, we measured the thorax temperature of individuals to the nearest 0.1°C with a micro‐thermocouple (BAT‐12 type, Physitemp Instruments, Clifton, NJ) directly after the flight test. We also counted the number of mites attached to individuals that were tested for their flight performance, as water mites have been shown to negatively influence flight ability (e.g., Nagel, Zanuttig, & Forbes, 2010) and mating success in male damselflies (Forbes & Robb, 2008), including the study species (Thompson, Hassall, Lowe, & Watts, 2011). Due to time constraints, we performed the flight test on a subset of individuals collected on a given day (total *N* = 338, see Table S1). Individuals with damaged wings were not tested.

### 2.3 Flight‐related physiological traits

We measured two flight‐related physiological traits of all collected males, based on the protocol by Swillen, De Block, and Stoks (2009): relative fat content and relative flight muscle mass. After removing wings and legs, we separated the head, thorax and abdomen using scissors. The thorax of each individual was placed separately in Eppendorf tubes, dried for 48 hr at 60°C and weighted to the nearest 0.01 mg using a microbalance. To extract the fat, we added 1.5 ml dichloromethane (99%) to the Eppendorf tubes, and placed them on an automatic shaker for 24 hr. After removal of the dichloromethane with the dissolved fat, we dried the body parts for another 48 hr at 60°C and weighed them again. Fat content was calculated by subtracting the dry masses before and after fat extraction. To obtain muscle mass, we added 1.5 ml NaOH (0.35 M) to the Eppendorf tubes to break down all muscle tissue. Finally, we placed the tubes on a shaker for 24 hr, and after removal of the NaOH with dissolved muscle tissue, we weighted the dried thorax without muscles. Flight muscle mass was quantified as the difference in dry mass of the thorax before and after this procedure; the flight muscles of odonates make up most of the thorax (Marden, 1989). Relative fat content was calculated as the ratio of fat mass in the thorax to body dry mass, whereas flight muscle ratio was calculated as the ratio of muscle mass to thorax dry mass.

### 2.4 Wing morphometrics

We quantified flight‐related wing characteristics using geometric morphometrics (Rohlf & Slice, 1990). We photographed the left hind wing of all males, and digitized the images in the tpsDig2 software (Rohlf, 2015). To capture a detailed wing shape, we placed seven landmarks along the wing outline where it is intersected by major wing veins, and five semi‐landmarks where intersections were not consistent between wings (Fig. S2). The landmark coordinates were imported into the software tpsRelw (Rohlf, 2015), where they were subjected to a generalized procrustes analysis in order to remove any non‐shape‐related differences due to variation in position, orientation and size (Rohlf & Slice, 1990). During this process, semi‐landmarks are allowed to slide along their tangent vectors to minimize the shape differences between specimens. After this step, the consensus conformation was compared with each specimen to generate shape variables termed partial warps. Finally, a principal component analysis was performed on these partial warp scores to compute relative warps. The first three relative warps explained 81.4% of the wing shape variation (relative warp 1 = 59.7%, relative warp 2 = 12.3%, relative warp 3 = 9.4%), and further analyses were conducted with these three relative warps. We visualized changes in wing shapes associated with increasing and decreasing relative warp scores by presenting transformation grids (Fig. S4) using the R package “geomorph” (Adams & Otárola‐Castillo, 2013).

Wing size was estimated as centroid size, the square root of the summed squared distances from each landmark to the geometric centre of each wing (Bookstein, 1991). As centroid size and body size are strongly correlated in damselflies (e.g., Outomuro & Johansson, 2011), we used wing centroid size as a proxy for body size (e.g., Outomuro, Adams, & Johansson, 2013; Outomuro et al., 2016). Finally, wing loading was measured as the ratio of total wet body mass to wing area, which was calculated as the polygon area enclosed by the landmarks.

### 2.5 Statistical analyses

To test for an effect of urbanization level (urban vs. rural) on the physiological (i.e., relative fat content and flight muscle ratio), wing morphological (i.e., centroid size, wing loading and three relative warps) and flight performance (i.e., flight endurance and speed) traits, we constructed separate linear mixed‐effect models per response variable. We included mating status (unmated vs. mated) and its interaction with urbanization level as an additional term in all models to control for potential variation in the traits due to mating status. All models had a normal error structure and the identity link and included population (nested within urbanization level) as a random effect.

To investigate for effects of the morphological and physiological traits on mating success, whether these effects are direct or indirectly mediated via flight performance, and differed between urban and rural males, we used a path analysis approach (Grace, 2006). This is a special case of structural equation modelling (SEM) that contains only observed variables. SEM allows to explore covariation patterns and causal relationships among variables (Grace, 2006). More specifically, we applied the generalized multilevel path analysis approach (Shipley, 2009) which allows for the implementation of non‐normally distributed (here binomial values of mating success), hierarchically structured data (here nested random effect of population), which cannot be applied in the traditional variance–covariance‐based SEM. This method has recently been successfully applied in several studies (e.g., Duffy, Lefcheck, Stuart‐Smith, Navarrete, & Edgar, 2016; Jing et al., 2015; Laliberte, Zemunik, & Turner, 2014; Theodorou et al., 2016).

We constructed an a priori path model (Fig. S3) based on previous research that investigated relationships between physiological and morphological traits and flight performance and/or mating success in damselflies (De Block & Stoks, 2007; Gosden & Svensson, 2008; Grether, 1996; Gyulavári et al., 2014, 2017; Outomuro et al., 2016; Steele, Siepielski, & McPeek, 2011; Swillen et al., 2009; Therry et al., 2014). As relative warps are unique shape variables that cannot be compared between studies, we could not predict their effect on the here used response variables. Hence, we included all possible paths between the three relative warps and the response variables (i.e., both flight performance traits and mating success) in the a priori path model. We also added the correlation between wing centroid size and wing loading to the path model. In addition, we added correlations between wing centroid size and the three relative warps to account for allometric effects.

To account for nonlinear relationships in the path model, we conducted an exploratory step where we tested for potentially interesting quadratic effects. We ran separate mixed‐effect models with either flight endurance, flight speed or mating success as response variable, and the linear term of a given trait as fixed effect. We then included the quadratic term of the trait as an additional fixed effect and compared the AIC values of the two models (i.e., model with linear vs. model with linear + quadratic fixed effect). We selected the quadratic relationship for the path model if the model with the quadratic term decreased the AIC score by 2 or more (i.e., resulted in a better supported model, Burnham & Anderson, 2002). Based on these exploratory analyses (data not shown), we included four additional paths to our a priori model: the quadratic term of relative fat content and centroid size with flight endurance as response variable, and the quadratic term of the second relative warp and centroid size with mating status as response variable.

We analysed the a priori path model by translating the path diagram into a set of linear mixed‐effect models (following Shipley, 2009): (i) flight endurance regressed against physiological and morphological traits; (ii) flight speed regressed against physiological and morphological traits; and (iii) mating success regressed against flight performance, physiological and morphological traits. In addition, we tested whether the following variables had an effect on flight performance by including them as covariates to the models: number of mites, body temperature, time of the flight test and time span between capture and flight test. We also tested whether number of mites had an influence on mating success. None of these covariates had a significant effect (all *p *>* *.13), and they were excluded from further analyses. All models incorporated population (nested within urbanization level) as random effect. This approach takes into account that individuals from the same populations are not independent replicates, thereby avoiding pseudoreplication. Flight performance variables were modelled with linear mixed‐effect models and had a normal error structure and the identity link, whereas mating success was modelled with generalized linear mixed‐effect models with a binomial error structure and a logit‐link function. Flight endurance, flight speed and wing centroid size were log‐transformed. All continuous variables were standardized (i.e., mean‐centred and divided by the standard deviation). As we had directional hypotheses for the relationship between flight performance and mating success (i.e., increased mating success with higher flight performance, Gyulavári et al., 2014; Therry et al., 2014; Gyulavári et al., 2017), we report one‐sided *p*‐values for the corresponding path coefficients. For the other path coefficients (i.e., paths going from physiological and morphological traits to flight performance and mating success), we report two‐sided *p*‐values.

To test whether the relationships between variables differed between the two urbanization levels, we then evaluated the a priori path model separately for urban and rural populations (see Minden et al., 2016; Theodorou et al., 2016 for a similar approach). In addition, we directly compared the path coefficients derived from the two separate path models (i.e., only urban vs. only rural) using the standard approach for comparing regression coefficients (Zar, 1999; see Dingemanse, Dochtermann, & Wright, 2010 for a similar approach). We compared path coefficients that had a *p *<* *.05 in either urban or rural models. For acquiring goodness‐of‐fit indices of the path models, we applied Shipley's d‐sep test (Shipley, 2002) which tests for any unrealized paths in the model, and provides Fisher's *C* statistics where the significance of unrealized paths is combined (see Shipley, 2009 for the calculations). By comparing the Fisher's *C* statistics to a χ^2^ distribution, we rejected or accepted each of the three models (i.e., models with *p *>* *.05 were accepted).

All analyses were performed using R version 3.2.3 (R Core Team 2013). We used the “lme4” package (Bates, Maechler, Bolker, & Walker, 2015) for mixed‐effect models, and the “piecewiseSEM” package (Lefcheck, 2016) for conducting path analyses. To control for multiple testing, we corrected for false discovery rate (FDR; Benjamini & Hochberg, 1995) when necessary.

## 3 RESULTS

**Figure 1:**
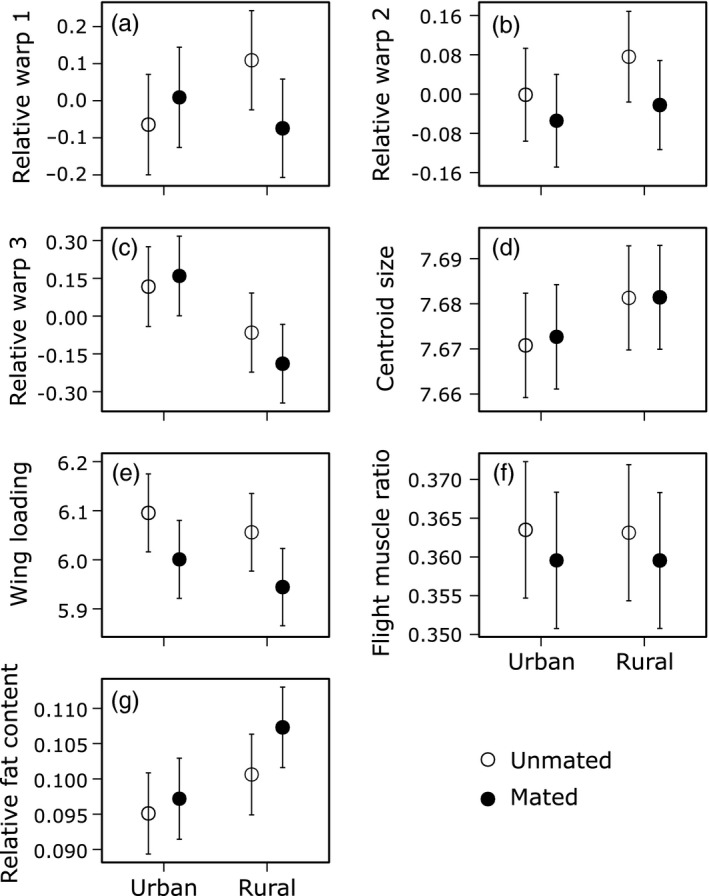
Mean (±1SE) values for flight‐related traits of *Coenagrion puella* males originating from urban and rural populations. (a) Relative warp 1, (b) relative warp 2, (c) relative warp 3, (d) centroid size (unitless measure), (e) wing loading (unitless measure), (f) flight muscle ratio and (g) relative fat content. Given are least‐squares means

**Figure 2:**
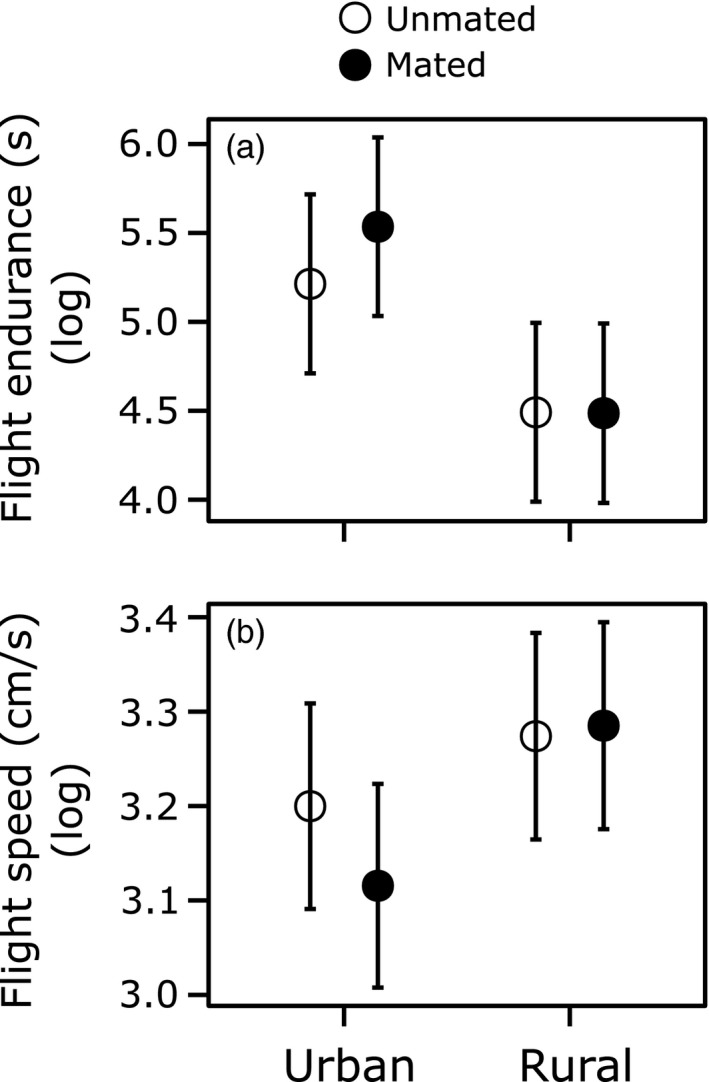
Mean (±1SE) values for flight performance traits of *Coenagrion puella* males originating from urban and rural populations. (a) Flight endurance, measured as total flight duration, and (b) flight speed. Given are least‐squares means

Urban males had higher positive scores for the relative warp 3 (Figure 1c; χ^2^ = 10.32, *df* = 1, *p *=* *.001, after FDR correction: *p *=* *.006). Moreover, urban males had a higher flight endurance (Figure 2a; χ^2^ = 32.73, *df* = 1, *p *<* *.001, after FDR correction: *p *<* *.001). None of the other measured traits differed significantly between urban and rural individuals (Figures 1 and 2; all *p *>* *.20), neither did urban–rural differences depend on the mating status (Urbanization level × Mating status: all *p *>* *.12).

**Figure 3:**
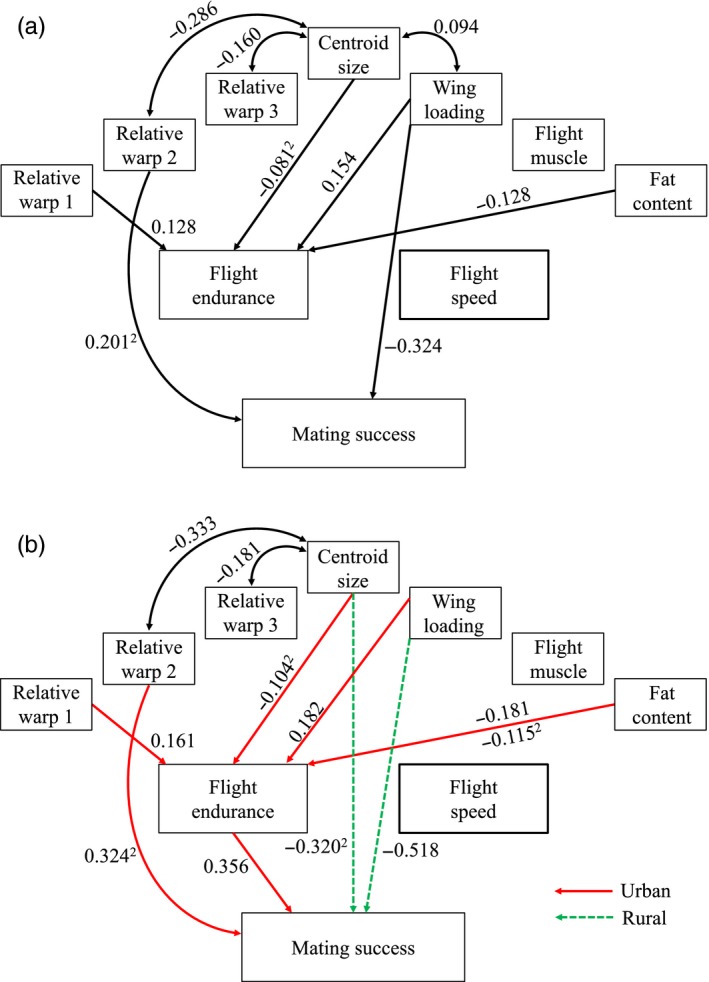
Path diagrams depicting the relationships between morphological and physiological traits, flight performance and mating success in urban and rural populations of the damselfly *Coenagrion puella*. Shown are path diagrams of (a) the “combined model”, where combined data of urban and rural populations were used, and (b) separate models for urban and rural populations. Double‐headed arrows indicate correlations between traits. Path coefficients are given next to the arrows (see text and Table S2 for details). To increase clarity, only significant (*p *<* *.05) paths are shown. Paths with quadratic terms are indicated with^2^

The a priori path model where urban and rural populations were combined (Figure 3a) was strongly supported by the data (*C*_16_ = 7.19, *p *=* *.969). Males with a lower fat content (*p *=* *.015), higher scores for relative warp 1 (slender wings, Fig. S4; *p *=* *.017) and higher wing loading scores (*p *=* *.007) had a higher flight endurance. Moreover, a significant negative quadratic effect of centroid size (*p *=* *.016) indicated that males with intermediate sized wings had higher flight endurance. Flight speed was not influenced by any measured physiological or morphological trait (all *p *>* *.20). Males with a lower wing loading (*p *=* *.014) had a higher mating success. Furthermore, a significant positive quadratic effect of relative warp 2 (*p *=* *.016) indicated that individuals with extreme high (short and broad wings) or low scores (long and slender wings) for relative warp 2 had a higher mating success (see also Fig. S4).

When we ran the a priori path model separately for urban and rural males (Figure 3b), the goodness‐of‐fit of the path models slightly increased (urban: *C*_16_ = 7.75, *p *=* *.956; rural: *C*_16_ = 8.49, *p *=* *.933), suggesting the covariation patterns differed between urban and rural males. The path model for urban males showed that higher values for relative warp 1 (*p *=* *.028) and wing loading (*p *=* *.019) were positively associated with flight endurance. As in the combined path model, flight endurance decreased with increasing fat content for urban males (linear effect: *p *=* *.009). Moreover, this decrease had a quadratic component (*p *=* *.014). A significant negative quadratic effect of centroid size (*p *=* *.026) indicated that males with intermediate sized wings had a higher flight endurance. Flight speed of urban males was not influenced by any physiological or morphological trait (all *p *>* *.13). A longer flight endurance (one‐sided *p *=* *.0305) increased the mating success of urban males. Mating success was affected by the quadratic term of relative warp 2 (*p *=* *.033), indicating that individuals with short and broad wings or long and slender wings had a higher mating success. The path model for rural males, on the other hand, revealed that no variable had a significant effect on flight performance. Instead, we found links to mating success that were not present in the path model for urban males (Figure 3): males with lower values for wing loading had higher mating success (*p *=* *.013), whereas a significant negative quadratic effect of centroid size (*p *=* *.035) indicated that males with intermediate centroid sizes had increased mating success.

Direct comparison confirmed several path coefficients to differ significantly between urban and rural models: the path going from the quadratic term of fat content (*t *=* *4.85, *df* = 32, *p *<* *.001) to flight endurance and paths from flight endurance (*t *=* *2.68, *df* = 32, *p* = .011), the quadratic term of relative warp 2 (*t *=* *2.75, *df* = 32, *p *=* *.009) and the quadratic term of centroid size (*t *=* *3.92, *df* = 32, *p *<* *.001) to mating success. Significance of the differences did not change after controlling for FDR (Table S3). This confirmed that flight endurance was only under sexual selection in urban populations.

## 4 DISCUSSION

As expected, urban damselfly populations were differentiated from rural populations by having a higher flight endurance. Moreover, urban individuals differed in their wing shape, as captured by higher scores for relative warp 3. Also the covariation patterns and causal relationships among the phenotypic traits, performance and mating success differed between urban and rural populations. Notably, in line with our prediction, the higher flight performance of urban males was reinforced by sexual selection. We discuss these findings in terms of potential adaptations to urban habitats.

### 4.1 Differentiation in performance and flight‐related traits between urban and rural populations

A key novelty of our study was the support for our prediction of a higher flight endurance in urban damselflies. This is in line with a recent interspecific study conducted in the same location (i.e., Flanders, Belgium), reporting that urban communities of carabid beetles consisted of species with better dispersal capacities compared to rural communities (Piano et al., 2017). While few intraspecific studies found indications of a better locomotor phenotype in urban populations (San Martin y Gomez & Van Dyck, 2012; Schoville et al., 2013), this was not general (Evans, Chamberlain, Hatchwell, Gregory, & Gaston, 2011), and no study directly compared flight performance between urban and rural populations. The latter is important as assumed phenotypic proxies for locomotor ability are not always empirically confirmed (e.g., Hanski, Breuker, Schops, Setchfield, & Nieminen, 2002). A higher flight endurance was expected given that urban ponds need to be colonized from source populations in rural areas (e.g., Evans et al., 2009) and habitats are more fragmented in urban areas (e.g., Cane et al., 2006; Luck & Wu, 2002). Given these characteristics of urban habitats, the selection for a better locomotor ability in more isolated urban populations resembles the selection forces experienced at invasion fronts and in fragmented landscapes. High dispersal abilities at invasion fronts are predicted by spatial sorting: the better dispersers will be the ones colonizing new habitats at the front, and assortative mating between fast‐dispersing individuals at invasion fronts results in further increase in dispersal ability (Shine et al., 2011). Similarly, fragmented landscapes are expected to drive increased locomotor performance, facilitating easier movement between isolated habitat patches (Dover & Settele, 2009; Van Dyck & Matthysen, 1999). The few studies that directly compared locomotor ability in these related contexts mostly were consistent with a higher locomotor ability at expansion fronts and in populations located in more fragmented landscapes. Indeed, cane toads evolved a higher locomotor ability at the invasion front (Shine, 2012), and flight endurance increased at the range expansion front in the damselfly *Coenagrion scitulum* (Swaegers et al., 2015). Further, the butterfly *Pieris brassicae* showed a higher flight performance with increasing landscape fragmentation (Ducatez et al., 2013). As we worked with field‐caught individuals, besides spatial sorting also plastic responses related to higher water temperatures in urban ponds may have been at work (for the study region, Brans et al., 2017). Yet, we recently showed that larvae of the damselfly *Ischnura elegans* reared at higher temperatures showed lower flight endurance in the adult stage (Arambourou, Sanmartín‐Villar, & Stoks, 2017), making our finding of a higher flight endurance in the urban populations occupying warmer ponds conservative.

Changes in flight‐related morphology associated with colonization events and landscape fragmentation is common in flying insects and is suggested to facilitate dispersal ability (Dover & Settele, 2009; Hill et al., 2011; Van Dyck & Matthysen, 1999). The direction of reported trait changes, however, is not consistent, especially so for wing size and shape. Longer and narrower wings, that is higher “wing aspect ratios,” have been reported for damselfly populations at range expansion fronts (*Calopteryx splendens*: Hassall, Thompson, & Harvey, 2009) and occupying fragmented habitats (*C. maculata*: Taylor & Merriam 1995). Studies with the speckled wood butterfly *Pararge aegeria* reported higher (Hughes, Dytham, & Hill, 2007) or lower wing aspect ratios (Hill, Thomas, & Blakeley, 1999) at edge populations compared to the core populations, whereas another study revealed no relationship between landscape fragmentation and wing aspect ratio in the same species (Merckx & Van Dyck, 2006). Moreover, larger wings in urban compared to rural populations have been documented in insects (the grasshopper *Chorthippus brunneus*: San Martin y Gomez & Van Dyck, 2012; the butterfly *Pieris rapae*: Schoville et al., 2013), and were suggested to result from selection for increased mobility. On the other hand, decreasing patch connectivity was associated with decreasing wing length in the blue‐winged grasshopper *Oedipoda caerulescens*, and was explained by lower investment in dispersal ability due to increasing cost of dispersal (Heidinger, Hein, & Bonte, 2010).

Given these conflicting patterns in flight‐related morphology associated with colonization events and landscape fragmentation, it may not be surprising that we have not found substantial differences in wing size and shape between urban and rural populations in the current study. Indeed, wing length (measured as centroid size) was not significantly different between urban and rural individuals. Further, the wing shape parameter relative warp 2, which can be considered analogous to wing aspect ratio (negative scores of relative warp 2 imply long and narrow wings; i.e., high wing aspect ratio), did not differ between urban and rural males. The only signal of urban–rural differentiation we detected was a higher score for relative warp 3 in urban compared to rural populations. However, this wing shape variable explained only ca. 9% of the total wing shape variation, hence only contributed little to the higher flight endurance in urban males. Furthermore, the subtle shape changes associated with relative warp 3, which are mainly focused on the wing apex (Fig. S4), make the interpretation of this wing shape variation difficult.

### 4.2 Differentiation in sexual selection on flight performance between urban and rural populations

In agreement with theory and previous empirical findings, we found positive sexual selection for higher flight endurance, yet only in the urban populations. For damselflies, this pattern has been demonstrated before, both directly by contrasting flight performance of mated and unmated males (Therry et al., 2014; Gyulavári et al., 2017; for the study species: Gyulavári et al., 2014), as well as indirectly by showing sexual selection on flight‐related traits (e.g., De Block & Stoks, 2007). Increased flight performance in scrambling mating systems is expected, as males with a higher mobility will encounter, hence mate with, more females (Kelly, Bussière, & Gwynne, 2008). As suggested (Husak & Fox, 2008) and empirically shown (Gyulavári et al., 2014; Therry et al., 2014), flight speed was not under sexual selection, confirming that flight speed is less important for scramble competitors. As expected, flight endurance was under sexual selection only in urban, and not in rural males. This somewhat matches the absence of sexual selection on maximum flight distance in a rural population of *Lestes sponsa* damselflies (Outomuro et al., 2016). The differentiation between population types in selection on flight endurance can be explained by the associated differentiation in mean flight endurance. Indeed, population differences in mean phenotypic traits may affect the direction and strength of selection, as populations will then span different parts of the species’ fitness function (Endler, 1986). For example, Steele et al. (2011) showed that temporal variation in mean body size of the damselfly *Enallagma aspersum* resulted in changes in the experienced fitness function at a given moment in time. For the present study, the shape of the overall fitness function across all populations reveals that while lower flight endurance is not under selection, flight endurance generates a mating advantage at higher endurance values (as present in the urban males). This confirms the shape of the fitness function for flight endurance as was previously reported for the study species (Fig. 1b in Gyulavári et al., 2014). We discuss the urban–rural differentiation in sexual selection on flight‐related traits and the differentiation in covariation patterns with flight performance in more detail in the Supporting Information.

Sexual selection is thought to accelerate adaptation to anthropogenic environments (Candolin & Heuschele, 2008), and at the same time anthropogenic environments have been shown to shape sexual selection (e.g., Candolin, Salesto, & Evers, 2007; Lane, Forrest, & Willis, 2011). Despite these insights, we have an extremely limited understanding of how urbanization, and even more general, habitat fragmentation can alter sexual selection (Murphy, Battocletti, Tinghitella, Wimp, & Ries, 2016). A notable exception in vertebrates is the divergence in sexually selected traits (male genitalia size and dorsal‐fin coloration) due to habitat fragmentation in *Gambusia* fish (Giery, Layman, & Langerhans, 2015; Heinen‐Kay, Noel, Layman, & Langerhans, 2014); however, in this system, the main factors driving this divergence were not fragmentation per se*,* but altered community composition and water chemistry. We also have poor knowledge whether sexual selection is aligned with natural selection and thereby reinforces natural selection in driving adaptive divergence between populations (Hendry, 2016). Our results provide the first evidence for a higher flight endurance in urban populations that was moreover associated with sexual selection for increased flight endurance in urban populations. Given that the increased flight endurance at edge populations of a related *Coenagrion* damselfly has been associated with genotypic differences (Swaegers et al., 2015), we might expect evolutionary divergence in flight performance in the derived urban damselfly populations. Together, this highlights the intriguing pattern that while urbanization shapes flight endurance and the associated sexual selection on endurance, the resulting sexual selection pattern reinforces the higher flight endurance in urban populations. Our results thereby provide unique proof in a single study system of the interplay between sexual selection and adaptation to anthropogenic environments (Candolin & Heuschele, 2008).

## DATA ARCHIVING STATEMENT

Data available from the Dryad Digital Repository: https://doi.org/10.5061/dryad.vs078.

## References

[eva12485-bib-0001] Adams, D. C. , & Otárola‐Castillo, E. (2013). Geomorph: An R package for the collection and analysis of geometric morphometric shape data. Methods in Ecology and Evolution, 4, 393–399.

[eva12485-bib-0002] Alberti, M. (2015). Eco‐evolutionary dynamics in an urbanizing planet. Trends in Ecology and Evolution, 30, 1–13. 25498964 10.1016/j.tree.2014.11.007

[eva12485-bib-0003] Alberti, M. , Correa, C. , Marzluff, J. M. , Hendry, A. P. , Palkovacs, E. P. , Gotanda, K. M. , … Zhou, Y. (2017). Global urban signatures of phenotypic change in animal and plant populations. Proceedings of the National Academy of Sciences of the United States of America, https://doi.org/10.1073/pnas.1606034114 10.1073/pnas.1606034114 PMC5576774 28049817

[eva12485-bib-0004] Arambourou, H. , Sanmartín‐Villar, I. , & Stoks, R. (2017). Wing shape‐mediated carry‐over effects of a heat wave during the larval stage on post‐metamorphic locomotor ability. Oecologia, 184, 279–291. 28238050 10.1007/s00442-017-3846-z

[eva12485-bib-0005] Baguette, M. , Legrand, D. , Fréville, H. , Van Dyck, H. , & Ducatez, S. (2012). Evolutionary ecology of dispersal in fragmented landscape In Clobert J., Baguette M., Benton T. G., & Bullock J. M. (Eds.), Dispersal ecology and evolution (pp. 381–391). Oxford: Oxford University Press.

[eva12485-bib-0006] Banks, M. , & Thompson, D. (1985). Lifetime mating success in the damselfly *Coenagrion puella* . Animal Behaviour, 33, 1175–1183.

[eva12485-bib-0007] Bates, D. , Maechler, M. , Bolker, B. , & Walker, S. (2015). Fitting linear mixed‐effects models using lme4. Journal of Statistical Software, 67, 1–48.

[eva12485-bib-0008] Benjamini, Y. , & Hochberg, Y. (1995). Controlling the false discovery rate: A practical and powerful approach to multiple testing. Journal of the Royal Statistical Society Series B, 57, 289–300.

[eva12485-bib-0009] Bergerot, B. , Merckx, T. , Van Dyck, H. , & Baguette, M. (2012). Habitat fragmentation impacts mobility in a common and widespread woodland butterfly: Do sexes respond differently? BMC Ecology, 12, 5. 22540674 10.1186/1472-6785-12-5 PMC3430564

[eva12485-bib-0010] Blanckenhorn, W. U. , Kraushaar, U. , & Reim, C. (2003). Sexual selection on morphological and physiological traits and fluctuating asymmetry in the yellow dung fly. Journal of Evolutionary Biology, 16, 903–913. 14635905 10.1046/j.1420-9101.2003.00587.x

[eva12485-bib-0011] Blanckenhorn, W. U. , Kraushaar, U. R. S. , Teuschl, Y. , & Reim, C. (2004). Sexual selection on morphological and physiological traits and fluctuating asymmetry in the black scavenger fly *Sepsis cynipsea* . Journal of Evolutionary Biology, 17, 629–641. 15149405 10.1111/j.1420-9101.2004.00693.x

[eva12485-bib-0012] Blanckenhorn, W. U. , Morf, C. , Mühlhäuser, C. , & Reusch, T. (1999). Spatiotemporal variation in selection on body size in the dung fly *Sepsis cynipsea* . Journal of Evolutionary Biology, 12, 563–576.

[eva12485-bib-0013] Bookstein, F. L. (1991). Morphometric tools for landmark data. Cambridge: Cambridge University Press.

[eva12485-bib-0014] Brans, K. I. , Govaert, L. , Engelen, J. M. T. , Gianuca, A. T. , Souffreau, C. , & De Meester, L. (2017). Eco‐evolutionary dynamics in urbanized landscapes: Evolution, species sorting and the change in zooplankton body size along urbanization gradients. Philosophical Transactions of the Royal Society B: Biological Sciences, 372, 20160030. 10.1098/rstb.2016.0030 PMC5182426 27920375

[eva12485-bib-0015] Burnham, K. P. , & Anderson, D. R. (2002). Model selection and multimodel inference: A practical information‐theoretic approach. New York, NY: Springer.

[eva12485-bib-0016] Candolin, U. , & Heuschele, J. (2008). Is sexual selection beneficial during adaptation to environmental change? Trends in Ecology and Evolution, 23, 446–452. 18582989 10.1016/j.tree.2008.04.008

[eva12485-bib-0017] Candolin, U. , Salesto, T. , & Evers, M. (2007). Changed environmental conditions weaken sexual selection in sticklebacks. Journal of Evolutionary Biology, 20, 233–239. 17210016 10.1111/j.1420-9101.2006.01207.x

[eva12485-bib-0018] Cane, J. H. , Minckley, R. L. , Kervin, L. J. , Roulston, T. H. , & Neal, W. M. (2006). Complex responses within a desert bee guild (Hymenoptera: Apiformes) to urban habitat fragmentation. Ecological Applications, 16, 632–644. 16711050 10.1890/1051-0761(2006)016[0632:crwadb]2.0.co;2

[eva12485-bib-0019] Cheptou, P.‐O. , Carrue, O. , Rouifed, S. , & Cantarel, A. (2008). Rapid evolution of seed dispersal in an urban environment in the weed *Crepis sancta* . Proceedings of the National Academy of Sciences of the United States of America, 105, 3796–3799. 18316722 10.1073/pnas.0708446105 PMC2268839

[eva12485-bib-0020] Conner, J. K. , & Hartl, D. L. (2004). A primer of ecological genetics. Sunderland, MA: Sinauer Associates.

[eva12485-bib-0021] Córdoba‐Aguilar, A. (2008). Dragonflies and damselflies: Model organisms for ecological and evolutionary research. Oxford: Oxford University Press.

[eva12485-bib-0022] Cote, J. , Bestion, E. , Jacob, S. , Travis, J. , Legrand, D. , & Baguette, M. (2017). Evolution of dispersal strategies and dispersal syndromes in fragmented landscapes. Ecography, 40, 56–73.

[eva12485-bib-0023] De Block, M. , & Stoks, R. (2007). Flight‐related body morphology shapes mating success in a damselfly. Animal Behaviour, 74, 1093–1098.

[eva12485-bib-0024] Dijkstra, K. D. B. , & Lewington, R. (2006). Field guide to the dragonflies of Britain and Europe. Gilingham: British Wildlife Publishing.

[eva12485-bib-0025] Dingemanse, N. J. , Dochtermann, N. , & Wright, J. (2010). A method for exploring the structure of behavioural syndromes to allow formal comparison within and between data sets. Animal Behaviour, 79, 439–450.

[eva12485-bib-0026] Dover, J. , & Settele, J. (2009). The influences of landscape structure on butterfly distribution and movement: A review. Journal of Insect Conservation, 13, 3–27.

[eva12485-bib-0027] Dubois, J. , & Cheptou, P.‐O. (2016). Effects of fragmentation on plant adaptation to urban environments. Philosophical Transactions of the Royal Society B: Biological Sciences, 372, 20160038. 10.1098/rstb.2016.0038 PMC5182434 27920383

[eva12485-bib-0028] Ducatez, S. , Baguette, M. , Trochet, A. , Chaput‐Bardy, A. , Legrand, D. , Stevens, V. , & Fréville, H. (2013). Flight endurance and heating rate vary with both latitude and habitat connectivity in a butterfly species. Oikos, 122, 601–611.

[eva12485-bib-0029] Duffy, J. E. , Lefcheck, J. S. , Stuart‐Smith, R. D. , Navarrete, S. A. , & Edgar, G. J. (2016). Biodiversity enhances reef fish biomass and resistance to climate change. Proceedings of the National Academy of Sciences of the United States of America, 113, 6230–6235. 27185921 10.1073/pnas.1524465113 PMC4896699

[eva12485-bib-0030] Endler, J. A. (1986). Natural selection in the wild. Princeton, NJ: Princeton University Press.

[eva12485-bib-0031] Evans, K. L. , Chamberlain, D. E. , Hatchwell, B. J. , Gregory, R. D. , & Gaston, K. J. (2011). What makes an urban bird? Global Change Biology, 17, 32–44.

[eva12485-bib-0032] Evans, K. L. , Gaston, K. J. , Frantz, A. C. , Simeoni, M. , Sharp, S. P. , McGowan, A. , … Hatchwell, B. J. (2009). Independent colonization of multiple urban centres by a formerly forest specialist bird species. Philosophical Transactions of the Royal Society B: Biological Sciences, 276, 2403–2410. 10.1098/rspb.2008.1712 PMC2691005 19364751

[eva12485-bib-0033] Forbes, M. R. , & Robb, T. (2008). Testing hypotheses about parasite‐mediated selection using odonate hosts In Córdoba‐Aguilar A. (Ed.), Dragonflies and damselflies: Model organisms for ecological and evolutionary research (pp. 175–188). New York, NY: Oxford University Press.

[eva12485-bib-0034] Giery, S. T. , Layman, C. A. , & Langerhans, R. B. (2015). Anthropogenic ecosystem fragmentation drives shared and unique patterns of sexual signal divergence among three species of Bahamian mosquitofish. Evolutionary Applications, 8, 679–691. 26240605 10.1111/eva.12275 PMC4516420

[eva12485-bib-0035] Goertzen, D. , & Suhling, F. (2013). Promoting dragonfly diversity in cities: Major determinants and implications for urban pond design. Journal of Insect Conservation, 17, 399–409.

[eva12485-bib-0036] Gosden, T. P. , & Svensson, E. I. (2008). Spatial and temporal dynamics in a sexual selection mosaic. Evolution, 62, 845–856. 18194470 10.1111/j.1558-5646.2008.00323.x

[eva12485-bib-0037] Grace, J. B. (2006). Structural equation modeling and natural systems. Cambridge: Cambridge University Press.

[eva12485-bib-0038] Grether, G. F. (1996). Sexual selection and survival selection on wing coloration and body size in the rubyspot damselfly *Hetaerina americana* . Evolution, 50, 1939–1948. 28565587 10.1111/j.1558-5646.1996.tb03581.x

[eva12485-bib-0039] Gyulavári, H. A. , Therry, L. , Dévai, G. , & Stoks, R. (2014). Sexual selection on flight endurance, flight‐related morphology and physiology in a scrambling damselfly. Evolutionary Ecology, 28, 639–654.

[eva12485-bib-0040] Gyulavári, H. A. , Tüzün, N. , Arambourou, H. , Therry, L. , Dévai, G. , & Stoks, R. (2017). Within‐season variation in sexual selection on flight performance and flight‐related traits in a damselfly. Evolutionary Ecology, 31, 21–36.

[eva12485-bib-0041] Hanski, I. , Breuker, C. J. , Schops, K. , Setchfield, R. , & Nieminen, M. (2002). Population history and life history influence the migration rate of female Glanville fritillary butterflies. Oikos, 98, 87–97.

[eva12485-bib-0042] Hassall, C. , Thompson, D. J. , & Harvey, I. F. (2009). Variation in morphology between core and marginal populations of three British damselflies. Aquatic Insects, 31, 187–197.

[eva12485-bib-0043] Heidinger, I. M. M. , Hein, S. , & Bonte, D. (2010). Patch connectivity and sand dynamics affect dispersal‐related morphology of the blue‐winged grasshopper *Oedipoda caerulescens* in coastal grey dunes. Insect Conservation and Diversity, 3, 205–212.

[eva12485-bib-0044] Heinen‐Kay, J. L. , Noel, H. G. , Layman, C. A. , & Langerhans, R. B. (2014). Human‐caused habitat fragmentation can drive rapid divergence of male genitalia. Evolutionary Applications, 7, 1252–1267. 25558285 10.1111/eva.12223 PMC4275096

[eva12485-bib-0045] Hendry, A. P. (2016). Eco‐evolutionary dynamics. Princeton, NJ: Princeton University Press.

[eva12485-bib-0046] Hill, J. K. , Griffiths, H. M. , & Thomas, C. D. (2011). Climate change and evolutionary adaptations at species’ range margins. Annual Review of Entomology, 56, 143–159. 10.1146/annurev-ento-120709-144746 20809802

[eva12485-bib-0047] Hill, J. K. , Thomas, C. D. , & Blakeley, D. S. (1999). Evolution of flight morphology in a butterfly that has recently expanded its geographic range. Oecologia, 121, 165–170. 28308556 10.1007/s004420050918

[eva12485-bib-0048] Hughes, C. L. , Dytham, C. , & Hill, J. K. (2007). Modelling and analysing evolution of dispersal in populations at expanding range boundaries. Ecological Entomology, 32, 437–445.

[eva12485-bib-0049] Husak, J. F. , & Fox, S. F. (2008). Sexual selection on locomotor performance. Evolutionary Ecology Research, 10, 213–228.

[eva12485-bib-0050] Jing, X. , Sanders, N. J. , Shi, Y. , Chu, H. , Classen, A. T. , Zhao, K. , … He, J.‐S. (2015). The links between ecosystem multifunctionality and above‐ and belowground biodiversity are mediated by climate. Nature Communications, 6, 8159. 10.1038/ncomms9159 PMC4569729 26328906

[eva12485-bib-0051] Kelly, C. D. , Bussière, L. F. , & Gwynne, D. T. (2008). Sexual selection for male mobility in a giant insect with female‐biased size dimorphism. The American Naturalist, 172, 417–423. 10.1086/589894 18651830

[eva12485-bib-0052] Kingsolver, J. G. , & Schemske, D. W. (1991). Path analyses of selection. Trends in Ecology and Evolution, 6, 276–280. 21232481 10.1016/0169-5347(91)90004-H

[eva12485-bib-0053] Laliberte, E. , Zemunik, G. , & Turner, B. L. (2014). Environmental filtering explains variation in plant diversity along resource gradients. Science, 345, 1602–1605. 25258078 10.1126/science.1256330

[eva12485-bib-0054] Lane, J. E. , Forrest, M. N. K. , & Willis, C. K. R. (2011). Anthropogenic influences on natural animal mating systems. Animal Behaviour, 81, 909–917.

[eva12485-bib-0055] Lefcheck, J. S. (2016). piecewiseSEM: Piecewise structural equation modeling in R for ecology, evolution, and systematics. Methods in Ecology and Evolution, 7, 573–579.

[eva12485-bib-0056] Luck, M. , & Wu, J. (2002). A gradient analysis of urban landscape pattern: A case study from the Phoenix metropolitan region, Arizona, USA. Landscape Ecology, 17, 327–339.

[eva12485-bib-0057] Marden, J. H. (1989). Bodybuilding dragonflies: Costs and benefits of maximizing flight muscle. Physiological Zoology, 62, 505–521.

[eva12485-bib-0058] Merckx, T. , & Van Dyck, H. (2006). Landscape structure and phenotypic plasticity in flight morphology in the butterfly *Pararge aegeria* . Oikos, 113, 226–232.

[eva12485-bib-0059] Minden, V. , Scherber, C. , Cebrián Piqueras, M. A. , Trinogga, J. , Trenkamp, A. , Mantilla‐Contreras, J. , … Kleyer, M. (2016). Consistent drivers of plant biodiversity across managed ecosystems. Philosophical Transactions of the Royal Society B: Biological Sciences, 371, 20150284. 10.1098/rstb.2015.0284 PMC4843704 27114585

[eva12485-bib-0060] Muñoz, P. T. , Torres, F. P. , & Megías, A. G. (2015). Effects of roads on insects: A review. Biodiversity and Conservation, 24, 659–682.

[eva12485-bib-0061] Murphy, S. M. , Battocletti, A. H. , Tinghitella, R. M. , Wimp, G. M. , & Ries, L. (2016). Complex community and evolutionary responses to habitat fragmentation and habitat edges: What can we learn from insect science? Current Opinion in Insect Science, 14, 61–65. 27436648 10.1016/j.cois.2016.01.007

[eva12485-bib-0062] Nagel, L. , Zanuttig, M. , & Forbes, M. R. (2010). Selection on mite engorgement size affects mite spacing, host damselfly flight, and host resistance. Evolutionary Ecology Research, 12, 653–665.

[eva12485-bib-0063] Outomuro, D. , Adams, D. C. , & Johansson, F. (2013). Wing shape allometry and aerodynamics in calopterygid damselflies: A comparative approach. BMC Evolutionary Biology, 13, 118. 23742224 10.1186/1471-2148-13-118 PMC3699362

[eva12485-bib-0064] Outomuro, D. , & Johansson, F. (2011). The effects of latitude, body size, and sexual selection on wing shape in a damselfly. Biological Journal of the Linnean Society, 102, 263–274.

[eva12485-bib-0065] Outomuro, D. , Söderquist, L. , Nilsson‐Örtman, V. , Cortázar‐Chinarro, M. , Lundgren, C. , & Johansson, F. (2016). Antagonistic natural and sexual selection on wing shape in a scrambling damselfly. Evolution, 70, 1582–1595. 27173835 10.1111/evo.12951

[eva12485-bib-0066] Parris, K. M. (2016). Ecology of urban environments. Chichester, West Sussex: Wiley‐Blackwell.

[eva12485-bib-0067] Phillips, B. L. , Brown, G. P. , Webb, J. K. , & Shine, R. (2006). Invasion and the evolution of speed in toads. Nature, 439, 803. 16482148 10.1038/439803a

[eva12485-bib-0068] Piano, E. , De Wolf, K. , Bona, F. , Bonte, D. , Bowler, D. E. , Isaia, M. , … Hendrickx, F. (2017). Urbanization drives community shifts towards thermophilic and dispersive species at local and landscape scales. Global Change Biology, https://doi.org/10.1111/gcb.13606 10.1111/gcb.13606 27997069

[eva12485-bib-0069] R Core Team (2013). R: A language and environment for statistical computing. Vienna: R Foundation for Statistical Computing.

[eva12485-bib-0070] Rohlf, F. J. (2015). TPS series. Department of Ecology and Evolution. Stony Brook University. Stony Brook, NY, USA. Retrieved from: http://life.bio.sunysb.edu/morph

[eva12485-bib-0071] Rohlf, F. J. , & Slice, D. (1990). Extensions of the Procrustes method for the optimal superimposition of landmarks. Systematic Biology, 39, 40–59.

[eva12485-bib-0072] San Martin y Gomez, G. , & Van Dyck, H. (2012). Ecotypic differentiation between urban and rural populations of the grasshopper *Chorthippus brunneus* relative to climate and habitat fragmentation. Oecologia, 169, 125–133. 22108853 10.1007/s00442-011-2189-4

[eva12485-bib-0073] Schoville, S. D. , Widmer, I. , Deschamps‐Cottin, M. , & Manel, S. (2013). Morphological clines and weak drift along an urbanization gradient in the butterfly, *Pieris rapae* . PLoS One, 8, 1–7. 10.1371/journal.pone.0083095 PMC3873920 24386146

[eva12485-bib-0074] Shine, R. (2012). Invasive species as drivers of evolutionary change: Cane toads in tropical Australia. Evolutionary Applications, 5, 107–116. 25568034 10.1111/j.1752-4571.2011.00201.x PMC3353345

[eva12485-bib-0075] Shine, R. , Brown, G. P. , & Phillips, B. L. (2011). An evolutionary process that assembles phenotypes through space rather than through time. Proceedings of the National Academy of Sciences of the United States of America, 108, 5708–5711. 21436040 10.1073/pnas.1018989108 PMC3078378

[eva12485-bib-0076] Shipley, B. (2002). Cause and correlation in biology: A user's guide to path analysis, structural equations and causal inference. Cambridge: Cambridge University Press.

[eva12485-bib-0077] Shipley, B. (2009). Confirmatory path analysis in a generalized multilevel context. Ecology, 90, 363–368. 19323220 10.1890/08-1034.1

[eva12485-bib-0078] Steele, D. B. , Siepielski, A. M. , & McPeek, M. A. (2011). Sexual selection and temporal phenotypic variation in a damselfly population. Journal of Evolutionary Biology, 24, 1517–1532. 21569154 10.1111/j.1420-9101.2011.02284.x

[eva12485-bib-0079] Sullivan, A. P. , Bird, D. W. , & Perry, G. H. (2017). Human behaviour as a long‐term ecological driver of non‐human evolution. Nature Ecology & Evolution, 1, 0065. 10.1038/s41559-016-0065 28812734

[eva12485-bib-0080] Swaegers, J. , Mergeay, J. , Van Geystelen, A. , Therry, L. , Larmuseau, M. H. D. , & Stoks, R. (2015). Neutral and adaptive genomic signatures of rapid poleward range expansion. Molecular Ecology, 24, 6163–6176. 26561985 10.1111/mec.13462

[eva12485-bib-0081] Swillen, I. , De Block, M. , & Stoks, R. (2009). Morphological and physiological sexual selection targets in a territorial damselfly. Ecological Entomology, 34, 677–683.

[eva12485-bib-0501] Taylor, P. D. , & Merriam, G. (1995). Wing morphology of a forest damselfly is related to landscape structure. Oikos, 73, 43–48.

[eva12485-bib-0082] Theodorou, P. , Radzevičiūtė, R. , Settele, J. , Schweiger, O. , Murray, E. T. , & Paxton, J. R. (2016). Pollination services enhanced with urbanisation despite increasing pollinator parasitism. Proceedings of the Royal Society B: Biological Sciences, 283, 20160561. 27335419 10.1098/rspb.2016.0561 PMC4936033

[eva12485-bib-0083] Therry, L. , Gyulavári, H. A. , Schillewaert, S. , Bonte, D. , & Stoks, R. (2014). Integrating large‐scale geographic patterns in flight morphology, flight characteristics and sexual selection in a range‐expanding damselfly. Ecography, 37, 1012–1021.

[eva12485-bib-0084] Thompson, D. J. , Hassall, C. , Lowe, C. D. , & Watts, P. C. (2011). Field estimates of reproductive success in a model insect: Behavioural surrogates are poor predictors of fitness. Ecology Letters, 14, 905–913. 21749601 10.1111/j.1461-0248.2011.01655.x

[eva12485-bib-0085] Travis, J. M. J. , & Dytham, C. (2002). Dispersal evolution during invasions. Evolutionary Ecology Research, 4, 1119–1129.

[eva12485-bib-0086] Van Dyck, H. , & Matthysen, E. (1999). Habitat fragmentation and insect flight: A changing “design” in a changing landscape? Trends in Ecology and Evolution, 14, 172–174. 10322528 10.1016/s0169-5347(99)01610-9

[eva12485-bib-0087] Van Petegem, K. H. P. , Boeye, J. , Stoks, R. , & Bonte, D. (2016). Spatial selection and local adaptation jointly shape life‐history evolution during range expansion. The American Naturalist, 188, 485–498. 10.1086/688666 27788346

[eva12485-bib-0088] Zar, J. H. (1999). Biostatistical analysis. Upper Saddle River, NJ: Prentice‐Hall.

