# Supplementary material for: Sexual selection reinforces a higher flight endurance in urban damselflies
Source: Evol Appl. 2017 May 11;10(7):694–703. doi: 10.1111/eva.12485 (PMC5511363; doi:10.1111/eva.12485)
Supplement: Supplementary file 1 [file EVA-10-694-s001.docx]

**Supplementary Information**

Sexual selection reinforces a higher flight endurance in urban damselflies

Nedim Tüzün, Lin Op de Beeck and Robby Stoks

**Table S1.** Coordinates and sampling information of the three urban and three rural populations of *Coenagrion puella* studied. Flight performance was quantified for only a subset of males.

| Population | Urbanisation level | Coordinates | Sampling period | Total number of males | | Total number of males in flight test | |
| --- | --- | --- | --- | --- | --- | --- | --- |
|  |  |  |  | unmated | mated | unmated | mated |
| Bierbeek | Rural | 50°47'27.87"N  4°42'12.17"E | 14-17.07.2013 | 47 | 50 | 25 | 25 |
| Bornem | Rural | 51°6'54.40"N  4°16'41.4"E | 24-25.07.2013 | 47 | 50 | 29 | 29 |
| Houwaart | Rural | 50°56'25.75"N  4°52'43.11"E | 15-19.07.2013 | 52 | 51 | 32 | 26 |
| Leuven | Urban | 50°52'26.17"N  4°41'48.69"E | 08-17.07.2013 | 49 | 53 | 28 | 29 |
| Mechelen | Urban | 51°3'15.43"N  4°26'40.65"E | 16-29.07.2013 | 40 | 40 | 27 | 29 |
| Oudenaarde | Urban | 50°50'45.78"N  3°36'57"E | 13-28.07.2013 | 49 | 48 | 29 | 30 |

**Table S2.** Standardized path coefficients of the ‘combined model’ (where combined data of urban and rural males were used), the ‘urban model’ (only including urban males), and the ‘rural model’ (only including rural males). Paths with quadratic terms are indicated with ^2^. Significant (*P* < 0.05) paths are given in bold. Note that we report one-sided *P* values for the “Mating success – flight endurance” path.

| Path | | Combined model | | | Urban model | | | Rural model | | |
| --- | --- | --- | --- | --- | --- | --- | --- | --- | --- | --- |
| Response | Predictor | Coefficient | SE | *P* | Coefficient | SE | *P* | Coefficient | SE | *P* |
| Flight endurance | Relative fat content | **-0.128** | **0.052** | **0.015** | **-0.181** | **0.068** | **0.009** | -0.133 | 0.084 | 0.138 |
|  | Relative fat content^2^ | -0.029 | 0.034 | 0.404 | **-0.115** | **0.046** | **0.014** | 0.05 | 0.055 | 0.37 |
|  | Relative warp 1 | **0.128** | **0.053** | **0.017** | **0.161** | **0.071** | **0.028** | 0.101 | 0.079 | 0.207 |
|  | Relative warp 2 | -0.001 | 0.052 | 0.987 | 0.066 | 0.072 | 0.36 | -0.067 | 0.076 | 0.391 |
|  | Relative warp 3 | 0.035 | 0.054 | 0.517 | 0.156 | 0.081 | 0.055 | -0.061 | 0.075 | 0.423 |
|  | Wing loading | **0.154** | **0.056** | **0.007** | **0.182** | **0.076** | **0.019** | 0.173 | 0.083 | 0.051 |
|  | Centroid size | -0.017 | 0.057 | 0.771 | 0.013 | 0.079 | 0.869 | -0.004 | 0.085 | 0.964 |
|  | Centroid size^2^ | **-0.081** | **0.033** | **0.016** | **-0.104** | **0.046** | **0.026** | -0.055 | 0.049 | 0.274 |
| Flight speed | Flight muscle ratio | 0.028 | 0.054 | 0.611 | 0.017 | 0.075 | 0.827 | 0.061 | 0.082 | 0.466 |
|  | Relative fat content | 0.047 | 0.055 | 0.407 | -0.052 | 0.075 | 0.484 | 0.145 | 0.084 | 0.107 |
|  | Relative warp 1 | -0.072 | 0.057 | 0.211 | -0.12 | 0.079 | 0.133 | -0.006 | 0.086 | 0.945 |
|  | Relative warp 2 | 0.006 | 0.053 | 0.913 | -0.013 | 0.075 | 0.86 | 0.004 | 0.078 | 0.957 |
|  | Relative warp 3 | -0.008 | 0.058 | 0.893 | -0.03 | 0.087 | 0.732 | 0.053 | 0.081 | 0.514 |
|  | Wing loading | -0.077 | 0.06 | 0.204 | -0.067 | 0.083 | 0.419 | -0.123 | 0.091 | 0.198 |
| Mating success | Flight endurance | 0.204 | 0.125 | 0.051 | **0.356** | **0.19** | **0.030** | 0.02 | 0.187 | 0.458 |
|  | Flight speed | -0.065 | 0.118 | 0.58 | -0.114 | 0.168 | 0.496 | -0.014 | 0.181 | 0.936 |
|  | Flight muscle ratio | -0.214 | 0.117 | 0.068 | -0.21 | 0.165 | 0.204 | -0.293 | 0.184 | 0.111 |
|  | Relative fat content | 0.163 | 0.119 | 0.17 | 0.076 | 0.163 | 0.641 | 0.275 | 0.191 | 0.149 |
|  | Relative warp 1 | -0.141 | 0.123 | 0.252 | -0.129 | 0.171 | 0.452 | -0.173 | 0.189 | 0.359 |
|  | Relative warp 2 | -0.131 | 0.124 | 0.293 | -0.162 | 0.184 | 0.377 | -0.105 | 0.184 | 0.568 |
|  | Relative warp 2^2^ | **0.201** | **0.083** | **0.016** | **0.324** | **0.152** | **0.033** | 0.096 | 0.104 | 0.355 |
|  | Relative warp 3 | -0.002 | 0.126 | 0.988 | 0.073 | 0.19 | 0.701 | -0.01 | 0.182 | 0.955 |
|  | Wing loading | **-0.324** | **0.131** | **0.014** | -0.27 | 0.183 | 0.14 | **-0.518** | **0.209** | **0.013** |
|  | Centroid size | 0.03 | 0.138 | 0.829 | 0.09 | 0.202 | 0.656 | -0.039 | 0.211 | 0.855 |
|  | Centroid size^2^ | -0.104 | 0.089 | 0.245 | 0.029 | 0.136 | 0.829 | **-0.32** | **0.152** | **0.035** |

**Table S3.** Direct pairwise comparison of path coefficients derived from the ‘urban model’ (including only urban males) and the ‘rural model’ (including only rural males). Paths with quadratic terms are indicated with ^2^. Paths that differed significantly (*P* < 0.05) are indicated in bold.

| Path | | Difference of path coefficient (Rural – Urban) | Pooled SE | *t* | df | *P* | *P* (after FDR correction) |
| --- | --- | --- | --- | --- | --- | --- | --- |
| Response | Predictor |  |  |  |  |  |  |
| Endurance | Relative fat content | 0.048 | 0.052 | 0.923 | 32 | 0.363 | 0.408 |
|  | Relative fat content^2^ | **0.165** | **0.034** | **4.853** | 32 | **< 0.001** | **< 0.001** |
|  | Relative warp 1 | -0.06 | 0.053 | 1.132 | 32 | 0.266 | 0.342 |
|  | Wing loading | -0.009 | 0.056 | 0.161 | 32 | 0.873 | 0.873 |
|  | Centroid size^2^ | 0.049 | 0.033 | 1.485 | 32 | 0.147 | 0.221 |
| Mating | Endurance | **-0.336** | **0.125** | **2.688** | 32 | **0.011** | **0.025** |
|  | Relative warp 2^2^ | **-0.228** | **0.083** | **2.747** | 32 | **0.010** | **0.025** |
|  | Wing loading | -0.248 | 0.131 | 1.893 | 32 | 0.067 | 0.121 |
|  | Centroid size^2^ | **-0.349** | **0.089** | **3.921** | 32 | **< 0.001** | **0.002** |

**Figure S1.** Location of the six study ponds. All ponds were located in Flanders, Belgium (inset).

**
Figure S2.** Location of landmarks used to describe wing shape in *Coenagrion puella* males. Numbers with asterisks correspond to semi-landmarks.

**Figure S3.** *A priori* path diagram, depicting the hypothesized relationships between morphological and physiological traits, flight performance and mating success in damselflies. In addition to linear terms, quadratic terms of relative fat content (with flight endurance as response variable), relative warp 2 (with mating success as response variable), and centroid size (with both flight endurance and mating success as response variable) were included to the *a priori* path model. Double-headed arrows indicate correlations between traits.

**Figure S4.** Wing shape variation in *Coenagrion puella* males, illustrated using thin-plate spline transformation grids. The first three relative warps (RW1-3) explained 81.4% of the wing shape variation. Negative (left panel) and positive (right panel) extremes for the three relative warps are shown. The most upper panel represents the mean wing shape. Transformation grids for RW2 and RW3 have been amplified (×3) for better illustration.

**Discussion**

*Differentiation in covariation patterns between urban and rural populations*

Flight performance was associated with various phenotypic traits, but the relationship strongly depended on the urbanisation level. Urban males with slender wings (positive scores for relative warp 1) had increased flight endurance. The mechanistic advantage of this wing type in terms of flight endurance cannot easily be linked to flight biomechanical studies as these typically measure wing aspect ratios instead of independent measures of wing length and width. Wings with intermediate centroid sizes resulted in a higher flight endurance, but only in urban males. This somewhat matches the absence of an effect of wing size (linear or quadratic) on maximum flight distance in a rural population of *Lestes sponsa* damselflies (Outomuro et al. 2016). The advantage of intermediate wing size in terms of flight endurance in urban males is not clear Wing loading was positively correlated with flight endurance in urban populations. Although this is in disagreement with the notion of the energetically more efficient low wing loading (Angelo and Slansky 1984), it has been reported in previous studies (e.g. Berwaerts et al. 2002). It is possible that all sampled populations from urban areas occupy habitats with abundant resources, compensating for the increased flight cost of higher wing loadings (Bartholomew and Casey 1978). Contrary to our expectation of a positive correlation of flight endurance and fat content (flight duration in insects depends on the amount of energy stores, Kaufmann et al. 2013), we found that fat content was associated with lower flight endurance in urban individuals, whereas there was no relationship in rural individuals (see also Gyulavári et al. 2017). Complex and opposite relationships between flight endurance and fat content may be partly explained by high fat contents allowing long flights, but at the same time long flights reducing the fat content (Arrese and Soulages 2010).

*Differentiation in sexual selection on flight-related traits between urban and rural populations*

Several flight-related traits were identified as targets of sexual selection and again, strongly depending on the population type. In urban populations, four traits (relative warp 1, centroid size, wing loading and relative fat content) were under indirect sexual selection as they contributed to mating success through their effect on flight endurance (discussed above). In addition, relative warp 2 directly shaped mating success. This took the form of disruptive sexual selection, indicating higher mating success for males with either long and narrow (high wing aspect ratio), or short and broad wings (low wing aspect ratio). This could indicate that these extreme wing shapes either contribute to an unmeasured aspect of flight performance which in turn increases mating success, or provide an advantage for mating success for other reasons. Instead, in rural populations we only found negative directional sexual selection for wing loading not mediated through flight endurance. Larger wings relative to body mass may indeed be advantageous for acquiring mates in scrambling mating systems, as it is energetically less costly (Angelo and Slansky 1984), hence allows allocating more time to searching females instead of food. Our study does not allow to explain why this would apply only to rural, and not urban males. Possibly, the higher air temperatures in urban areas (the so-called “urban heat island” effect: Arnfield 2003) create more favourable flight conditions, thereby reducing costs of long flight for urban males. Similarly, the negative directional sexual selection for wing loading in rural males could be a compensation for reduced wing beat frequency at the typically cooler temperatures of rural areas (e.g. Merckx et al. 2006). Finally, we detected stabilising sexual selection on wing size (also proxy for body size) only in rural populations. This pattern has previously been shown in damselflies (Stoks 2000, Thompson and Fincke 2002; Gyulavári et al. 2017). Interestingly, a study with the damselfly *Ischnura elegans* revealed that the selection on male body size can take almost any form, depending on the sampled population and the year of sampling (Gosden and Svensson 2008). The authors suggest this was driven by densities of the female colour morphs (which also occurs in the study species), and female body sizes. We did not find any urbanisation-related difference in female body size (unpublished data), yet we have no data on the density of female colour morphs from the sampled locations, which could potentially explain the spatial variation in selection on body size found in the present study.

**References**

Angelo, M. J., and F. Slansky. 1984. Body building by insects: trade-offs in resource allocation with particular reference to migratory species. The Florida Entomologist 67:22–41.

Arnfield, A. J. 2003. Two decades of urban climate research: a review of turbulence, exchanges of energy and water, and the urban heat island. International Journal of Climatology 23:1–26.

Arrese, E. L., and J. L. Soulages. 2010. Insect fat body: energy, metabolism, and regulation. Annual Review of Entomology 55:207–225.

Bartholomew, G. A., and T. M. Casey. 1978. Oxygen consumption of moths during rest, pre-flight warm-up, and flight in relation to body size and wing morphology. Journal of Experimental Biology 76:11–25.

Berwaerts, K., H. Van Dyck, and P. Aerts. 2002. Does flight morphology relate to flight performance? An experimental test with the butterfly *Pararge aegeria*. Functional Ecology 16:484–491.

Gosden, T. P., and E. I. Svensson. 2008. Spatial and temporal dynamics in a sexual selection mosaic. Evolution 62:845–856.

Gyulavári, H. A., N. Tüzün, H. Arambourou, L. Therry, G. Dévai, and R. Stoks. 2017. Within-season variation in sexual selection on flight performance and flight-related traits in a damselfly. Evolutionary Ecology 31:21–36

Kaufmann, C., C. Reim, and W. U. Blanckenhorn. 2013. Size-dependent insect flight energetics at different sugar supplies. Biological Journal of the Linnean Society 108:565–578.

Merckx, T., B. Karlsson, and H. Van Dyck. 2006. Sex- and landscape-related differences in flight ability under suboptimal temperatures in a woodland butterfly. Functional Ecology 20:436–441.

Stoks, R 2000. Components of lifetime mating success and body size in males of a scrambling damselfly. Animal Behaviour 59:339-348.

Thompson, D. J., and O. M. Fincke. 2002. Body size and fitness in Odonata: stabilising selection and a meta-analysis too far? Ecological Entomology 27:378–384.
